# Flow cytometry-assisted rapid isolation of recombinant *Plasmodium berghei* parasites exemplified by functional analysis of aquaglyceroporin

**DOI:** 10.1016/j.ijpara.2012.10.006

**Published:** 2012-12

**Authors:** Sanketha Kenthirapalan, Andrew P. Waters, Kai Matuschewski, Taco W.A. Kooij

**Affiliations:** a Max Planck Institute for Infection Biology, Parasitology Unit, Berlin, Germany; b Wellcome Trust Centre for Molecular Parasitology, Glasgow Biomedical Research Centre, University of Glasgow, Glasgow, Scotland, UK; c Institute of Biology, Humboldt University, Berlin, Germany

**Keywords:** *Apicomplexa*, Aquaglyceroporin, Cloning, Experimental genetics, Flow cytometry, *Plasmodium berghei*

## Abstract

The most critical bottleneck in the generation of recombinant *Plasmodium berghei* parasites is the mandatory in vivo cloning step following successful genetic manipulation. This study describes a new technique for rapid selection of recombinant *P. berghei* parasites. The method is based on flow cytometry to isolate isogenic parasite lines and represents a major advance for the field, in that it will speed the generation of recombinant parasites as well as cut down on animal use significantly. High expression of GFP during blood infection, a prerequisite for robust separation of transgenic lines by flow cytometry, was achieved. Isogenic recombinant parasite populations were isolated even in the presence of a 100-fold excess of wild-type (WT) parasites. Aquaglyceroporin (AQP) loss-of-function mutants and parasites expressing a tagged AQP were generated to validate this approach. *aqp*^−^ parasites grow normally within the WT phenotypic range during blood infection of NMRI mice. Similarly, colonization of the insect vector and establishment of an infection after mosquito transmission were unaffected, indicating that *AQP* is dispensable for life cycle progression in vivo under physiological conditions, refuting its use as a suitable drug target. Tagged AQP localized to perinuclear structures and not the parasite plasma membrane. We suggest that flow-cytometric isolation of isogenic parasites overcomes the major roadblock towards a genome-scale repository of mutant and transgenic malaria parasite lines.

## Graphical abstract

**Graphical abstract:**

**Figure:**
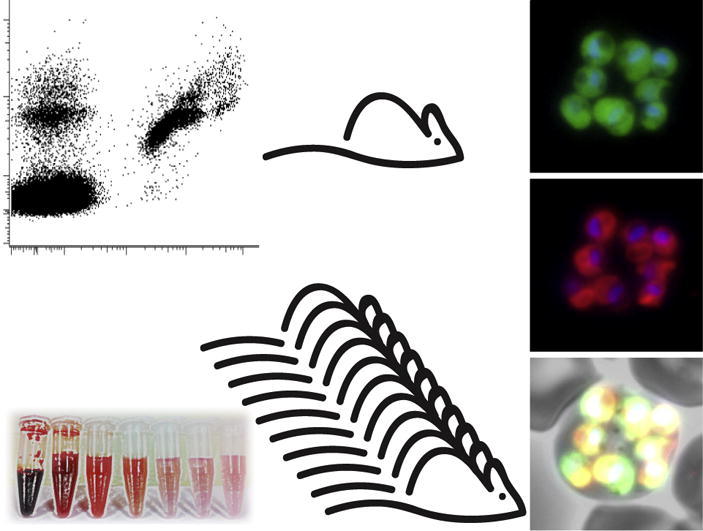

**Highlights:**

► Rapid, robust isolation of recombinant *Plasmodium berghei* lines by flow cytometry. ► Reduction by >80% of animal use for the generation of mutant parasite lines. ► *Plasmodium berghei* aquaglyceroporin is dispensable for all life cycle stages. ► Tagged aquaglyceroporin localizes to perinuclear structures inside the parasite.

## 1 Introduction

Experimental reverse genetics in the rodent malaria model parasite *Plasmodium berghei* is a well-established approach that has fundamentally changed our understanding of the molecular biology of malaria parasites (de Koning-Ward et al., 2000). A major advantage of studying *P. berghei* is the accessibility of the complete life cycle, including experimental infections of the mammalian host. In addition, it is the most straightforward of all *Plasmodium* spp. to genetically manipulate (Janse et al., 2011).

A major bottleneck in the generation of mutant parasite lines is the obligate in vivo cloning step. This is being performed by i.v. injection of single infected erythrocytes obtained by serial dilutions (Ménard and Janse, 1997). For every in vivo cloning experiment, approximately 10 naïve mice are needed. A typical functional analysis of a selected gene often includes a transgenic line expressing a fusion to a fluorescent and/or epitope tag in parallel with a loss-of-function mutant (Haussig et al., 2011). Furthermore, it has been suggested that knockout (KO) mutants originating from at least two independent transfection experiments should be examined (Goldberg et al., 2011). Thus, the number of mice required just to clone mutant parasite lines following successful transfection cumulates to >30 when using the established method of limiting dilution. Housing, continuous care and handling of these high numbers of experimental animals per *Plasmodium* target gene have essentially prevented progress towards a genome-scale repository for community-wide phenotyping of recombinant malaria parasites.

The use of flow cytometry to isolate an isogenic parasite mutant line has the potential to dramatically reduce the number of mice required. However, methods and plasmids developed thus far have yielded inconsistent results. In particular, all published KO parasites thus far have been generated using the traditional in vivo cloning procedure (Janse et al., 2011). Despite recent advances in the generation of improved *P. berghei* targeting vectors (Pfander et al., 2011) the generation of pure, recombinant parasites remains the most important roadblock. We recently developed the multi-purpose *Berghei* Adaptable Transfection plasmid (pBAT), which includes a high-expressing GFP cassette (Kooij et al., 2012). Here, we establish an improved and standardized strategy for the generation of isogenic mutant parasite lines based on the GFP cassette in pBAT.

Two complementary strategies were used to validate the novel approach, namely targeted deletion and fluorescent tagging, to study the developmental role(s) of *P. berghei* aquaglyceroporin (AQP, **PBANKA_091560**). This gene was selected because previous work indicated a prominent growth defect during asexual blood stage development of recombinant *P. berghei* parasites lacking *AQP* (Promeneur et al., 2007). However, no further analysis of *AQP* function during mosquito and pre-erythrocytic development has been performed. A recent high-resolution crystal structure revealed the dual specificity of *Plasmodium falciparum* AQP (*Pf*AQP) for water and glycerol, setting this protein apart from its close homologs (Newby et al., 2008). Therefore, a potential vital function in stages other than during asexual blood infection might offer novel drug intervention approaches. Moreover, localization studies using polyclonal anti-peptide antisera yielded inconsistent results and interpretations, with the localization of AQP described as either intracellular and patchy (Hansen et al., 2002) or in the parasite plasma membrane (Promeneur et al., 2007). Hence, fluorescent tagging of endogenous AQP was expected to clarify whether the channel is parasite surface-expressed and, hence, contributes to solute uptake from host cells or participates in their accumulation in parasite organelles.

## 2 Materials and methods

### 2.1 Experimental animals

This study was carried out in strict accordance with the German ‘Tierschutzgesetz in der Fassung vom 22. Juli 2009’ and the Directive 2010/63/EU of the European Parliament and Council ‘On the protection of animals used for scientific purposes’. The protocol was approved by the ethics committee of the Berlin, Germany, state authority (‘Landesamt für Gesundheit und Soziales Berlin’, permit number G0469/09). C57BL/6 mice were used for sporozoite infections. One blood stage infection experiment was performed in Swiss-Webster mice. All other parasite infections were conducted with NMRI mice.

### 2.2 Generation of aqp^−^ and aqp::tag parasites

Parasites with their *AQP* disrupted or tagged C-terminally were generated using the standard gene replacement strategy (Janse et al., 2006a). First, a 526 bp fragment of the 3′ untranslated region (UTR) was amplified by PCR from genomic DNA (gDNA) using the primer combination AQP-F5-*Avr*II and AQP-R4-*Kpn*I and cloned into the pBAT-SIL6 vector (Kooij et al., 2012) using *Avr*II and *Kpn*I restriction digestion to generate the intermediate construct pAQP-IM.

For the generation of the *AQP* disruption construct, pAQP-KO, a 557 bp fragment of the 5′UTR was amplified from gDNA using the primer combination AQP-F2-*Sac*II and AQP-R1-*Eco*RI. This fragment was subcloned, using *Eco*RI and *Sac*II, into an intermediate pBAT-derived vector. Subsequently, it was released by cleavage with *Hpa*I and *Sac*II, and cloned into pAQP-IM using *Sac*II and *Pvu*II. For the tagging construct, pAQP-tag, a 527 bp fragment of the C-terminal coding region of *AQP* was amplified from gDNA using the gene-specific primers, AQP-F4-*Sac*II and AQP-R3-*Psh*AI, and fused in frame to the mCherry-3xMyc tag of pAQP-IM using *Sac*II and *Hpa*I. The two resulting plasmids, pAQP-KO and pAQP-tag, were linearized with *Sca*I and *Sal*I and transfected into WT *P. berghei* ANKA strain parasites.

### 2.3 Flow-cytometric isolation of recombinant parasites

The flow cytometry protocol was developed starting with flow-cytometric parameters as described for the isolation of the GFPcon parasite line (Janse et al., 2006b). A number of significant modifications were introduced for efficient isolation of pure isogenic populations. Between days 7 and 9 after transfection, when parasitemia of the parental population was still <1%, one drop of tail blood was collected and resuspended in 1 ml of Alsever’s solution (Sigma–Aldrich, Germany) in order to minimize agglutination of the blood. The samples were passed through 30 μm CellTrics filters (Partec, Germany) to further remove cell aggregates. All sorting experiments were performed on a BD Biosciences FACSDiva using a purity sort-mask at a sorting speed of 30,000 events per second. Excitation of GFP was done at a wavelength of 488 nm, while fluorescence was detected using a band pass filter of 530/30 nm with the photomultiplier tube voltage set to its maximum sensitivity. Forward and sideward scatter gating was used to exclude small particles (such as blood platelets and debris) as well as overly large cells (predominantly leukocytes). The remaining population mainly consisted of erythrocytes and was gated for high-expressing GFP (GFP^hi^) but low-expressing phycoerythrin (PE^lo^) cells, thus excluding autofluorescent cells with equal GFP and PE levels, 500 of which were collected in 200 μl of RPMI complemented with 20% FCS. To optimize cell survival, the deflection plate voltage and drop charge were set such that sorted cells were collected at the bottom of the tube. In addition, the sides of the collection tube were pre-wetted with culture medium. Following sorting, the collection tube was washed with 800 μl of RPMI/FCS. The entire flow cytometry protocol was performed at room temperature. Naïve recipient NMRI mice were injected i.v. with 100 μl cell suspension containing 50 GFP^hi^ cells. At a parasitemia of >1%, mice were bled, parasite stabilates were frozen, and parasite gDNA was isolated.

### 2.4 Genotyping of aqp^−^ and aqp::tag parasites

Correct integration of the transfection vectors in the mutant parasite lines and absence of contaminating WT parasites following flow cytometric isolation were confirmed using the following specific primer combinations: AQP-F1 and 5′HSP70rev (5′ integration of pAQP-KO, 1,027 bp), AQP-F3 and mCherryRev (5′ integration of pAQP-tag, 675 bp), 5′DHFRrev and AQP-R5 (3′ integration of both vectors, 1,352 bp), AQP-F1 and AQP-R2 (5′ WT for the control of *aqp*^−^ only, 983 bp), and AQP-F3 and AQP-R5 (3′ WT, 1,337 bp). All primer sequences are listed in Supplementary Table S1.

Both independent *aqp*^−^ lines were analyzed by Southern blot using the PCR DIG Probe Synthesis kit and the DIG Luminescent Detection kit (Roche, Germany), according to the manufacturer’s protocol. The 5′ and 3′ probes were amplified using primers AQP-F2-*Sac*II and AQP-R1-*Eco*RI, and AQP-F5-*Avr*II and AQP-R4-*Kpn*I, respectively, and annealed to *Nde*I restriction-digested gDNA resulting in bands of 2.2 and 5.7 kb (*aqp*^−^) and 1.5 and 0.8 kb (WT).

### 2.5 Analysis of the *Plasmodium* life cycle progression

To compare blood stage development of *aqp*^−^ and WT parasites, 1,000 infected erythrocytes were injected i.v. into naïve recipient NMRI or Swiss-Webster mice. The progress of the infection was monitored by daily microscopic examination of Giemsa-stained thin blood smears.

Mosquito stage development was analyzed using standard techniques (Vanderberg, 1975). Mice were infected by i.v. injection of 10^7^ parasitized erythrocytes. Prior to feeding mosquitos on infected mice 3 days later, a drop of tail blood was analyzed under a conventional light microscope to determine the exflagellation activity per μl of blood. To determine infectivity and the number of midgut sporozoites, mosquitos were dissected on day 14 after feeding. The number of salivary gland sporozoites was determined on day 17. To determine the sporozoite infectivity to mice, 10,000 salivary gland sporozoites were isolated on day 17 and injected i.v. into naïve recipient C57BL/6 mice. Alternatively, naïve recipient C57BL/6 mice were infected by natural bite of 12 infected mosquitoes. Patency was determined by examination of daily Giemsa-stained thin blood smears.

Liver stages of *aqp::tag* parasites were cultured in vitro and analyzed as previously described (Haussig et al., 2011). At 48 h p.i., hepatoma cultures were fixed and incubated with monoclonal mouse anti-*P. berghei* heat shock protein 70 (HSP70) (1:300 dilution) (Tsuji et al., 1994) and rat anti-mCherry (1:500 dilution, Chromotek, Germany) antibodies. Bound antibodies were detected using goat anti-mouse/rat IgG Alexa Fluor 488/546 conjugated antibodies (1:1,000 dilution, Invitrogen, Germany). Nuclei were visualized with the DNA-dye, Hoechst 33342 (Invitrogen).

### 2.6 Image acquisition

All images were recorded on a Leica DMR epifluorescence microscope and processed minimally with ImageJ (http://rsb.info.nih.gov/ij/). Nuclei of live parasites were visualized with the DNA-dye, Hoechst 33342 (Invitrogen).

## 3 Results

### 3.1 Establishment of flow cytometry conditions for the isolation of recombinant parasites

**Fig. 1:**
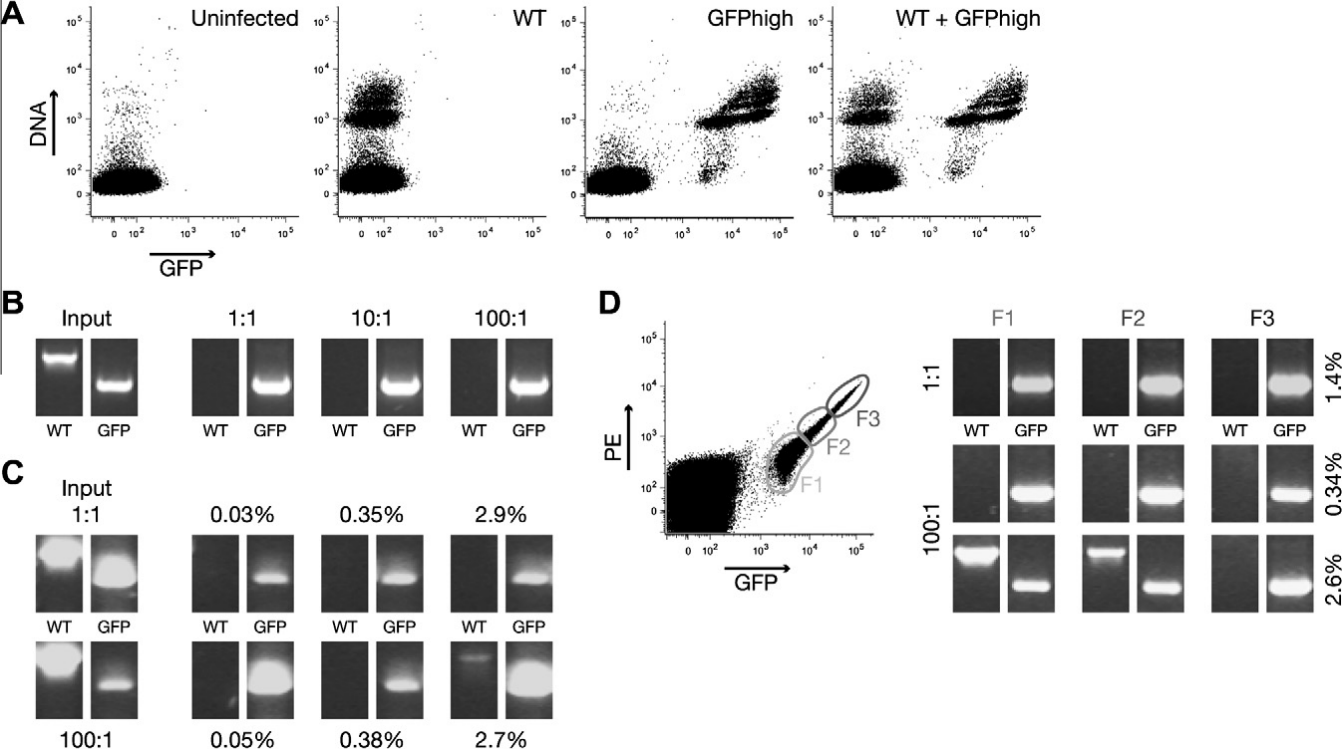
Robust isolation of GFPhigh from non-fluorescent wild type (WT) *P. berghei* parasites. (A) Scatter plots of uninfected blood and blood infected with WT, GFPhigh, and a mixed parasite population. DNA was stained with Hoechst 33342. GFPhigh parasites expressing GFP under the control of the *P. berghei* heat shock protein 70 (*PbHSP70*) promoter sequence are clearly separated from non-fluorescent WT parasites. Hoechst^hi^ GFP^lo^ cells appear in the scatter plot of uninfected blood and blood infected with pure GFPhigh parasites, which probably correspond to mouse leukocytes. (B) Blood from two mice infected with either WT or GFPhigh parasites (input) was mixed in three different ratios and sorted using standard conditions. Naïve recipient mice were infected with 50 GFP^hi^ cells. When the parasitemia had reached >1% parasite DNA was isolated and a diagnostic PCR was done to determine the presence of WT (using primers SIL6F and SIL6R, 1,315 bp) and/or GFPhigh (using primers SIL6F and mCherryRev, 783 bp) parasites (see Supplementary Table S1 for primer sequences). Note that despite a 100-fold excess of WT parasites, pure GFPhigh parasites were confidently isolated. (C) Two donor mice were infected with WT and GFPhigh parasites in the ratios indicated (input). At the indicated parasitemias, 50 GFP^hi^ cells were isolated and injected into naïve recipient mice. Note that only at higher parasitemia and in the presence of a 100-fold excess of WT parasites, minor WT populations were detectable. (D) Two donor mice were infected with WT and GFPhigh parasites in the ratios indicated. At the indicated parasitemias, 50 GFP^hi^ cells were isolated using three different gates (F1–3) and injected into naïve recipient mice.

To establish a robust isolation procedure for GFP^hi^ parasites from non-fluorescent WT parasites, an independent GFP^hi^ parasite line was generated, analogous to the generation of the Bergreen and related parasite lines (Kooij et al., 2012). WT parasites were transfected with *Apa*LI-linearized pBAT-SIL6, which integrates in a silent intergenic locus on *P. berghei* chromosome 6 and introduces a recyclable, drug-selectable cassette and a high-expressing GFP cassette under the control of the *P. berghei HSP70*. This independent line as well as the original Bergreen and 6G parasite lines were used as GFP^hi^ control parasites that grow with WT parasite kinetics (Kooij et al., 2012) and will commonly be referred to as GFPhigh. When tested by flow cytometry, these parasites showed consistently high levels of GFP expression, resulting in a clear separation from non-infected erythrocytes and leukocytes present in mouse blood (Fig. 1A). This pattern in flow-cytometric analysis was clearly different from that obtained with WT parasite-infected blood. When blood from a mixed infection was analyzed, two distinct populations, Hoechst^hi^ GFP^lo^ and Hoechst^hi^ GFP^hi^ reflecting WT and GFPhigh parasites, respectively, were readily distinguishable (Fig. 1A).

To test whether the sorting procedure itself was reliable and to demonstrate the absence of occasional non-fluorescent WT parasite-infected cells, blood from mice infected with either WT or GFPhigh parasites was mixed in (i) equal amounts, or with a (ii) 10-fold, or (iii) 100-fold excess of WT parasite-infected cells. After flow-cytometric sorting of the blood mixtures, 50 GFP^hi^ cells were injected i.v. into naïve recipient mice. Encouragingly, only GFPhigh, and no WT, parasite gDNA was detectable (using PCR) in recipient infected mice, irrespective of the excess of WT parasites (Fig. 1B).

To extend this finding, the time course of mixed infections was investigated over several days. When equal amounts of WT and GFPhigh parasites were present in the donor population, GFPhigh could be confidently sorted from WT parasites, even at high parasitemia (2.9%). In good agreement with the efficient sorting of in vitro mixed parasites (Fig. 1B), a 100-fold excess of WT parasites did not interfere with the successful sorting of GFPhigh parasites from a mixed infection (Fig. 1C). However, at a high parasitemia (2.7%), a minor WT contamination was observed in the sorted population (Fig. 1C), most likely due to inevitable sorting of a double-infected erythrocyte, a frequent aspect in highly infected blood.

Finally, whether selection of a specific subpopulation of GFP^hi^ cells might be used to further optimize sorting of an isogenic population was tested. Blood from mice infected with equal amounts of WT and GFPhigh parasites was sorted without detectable WT parasite contamination, irrespective of the gate used (Fig. 1D). Specific gating of the most prominent GFP^hi^ population, termed F3, even enabled the isolation of a population of pure GFPhigh parasites when applied at a high parasitemia (2.6%) of a mixed population with 100-fold WT excess. Together, the results demonstrate that flow cytometry followed by i.v. injection of 50 infected erythrocytes permits the isolation of isogenic parasites from rare populations ex vivo.

### 3.2 Optimization of transfection DNA preparation to eliminate episomal transfection

**Fig. 2:**
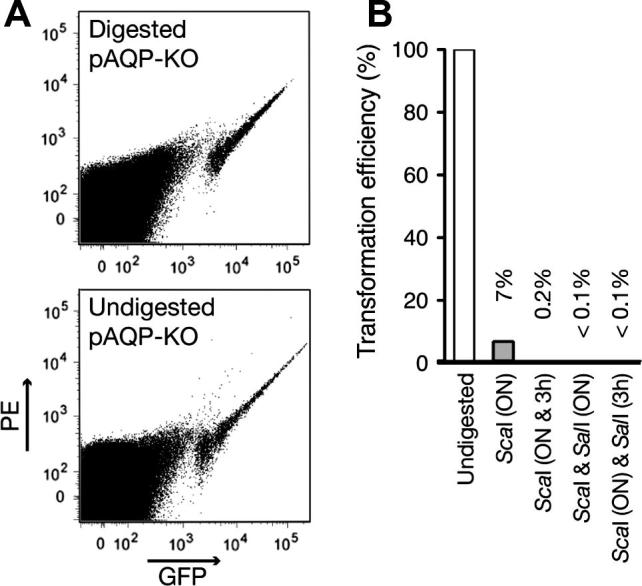
Optimization of transfection DNA preparation to minimize episomal transfection. (A) Scatter plots of parental *P. berghei* populations transfected with digested and undigested aquaglyceroporin disruption construct (pAQP-KO) show only marginal differences. The non-autofluorescent (equal GFP and phycoerythrin (PE) levels) GFP^hi^ population of parasites transfected with undigested DNA shows parasites with both slightly lower and with slightly higher GFP levels. (B) To eliminate the transfection of episomal DNA, ideally the plasmid DNA should be digested 100% to completion. *Escherichia coli* XL1-Blue cells were transformed with 300 ng of transfection DNA and the number of colonies was quantified relative to cells transformed with undigested plasmid DNA. Standard overnight (ON) digestion with a single restriction endonuclease (*Sca*I) yielded 7%, addition of enzyme for the final 3 h of digestion resulted in 0.2%, and the addition of a second restriction endonuclease (*Sal*I) either overnight or for the final 3 h produced ⩽0.1% colonies.

A latent problem when using flow cytometry for the generation of an isogenic population of mutant parasites is the potential contamination with episomally transfected parasites. Therefore, the possibility to eliminate this group of parasites based on GFP-intensity was investigated. One could argue that multiple plasmid copies might give rise to even higher GFP levels than are present in parasites that have stably integrated one GFP copy into their genomes. To test this, parasites were transfected with a targeting vector, termed pAQP-KO (see below), which was either digested twice or left undigested (Fig. 2A). Although a minor fraction of parasites with abnormally high levels of GFP was observed in the parasites transfected with undigested, episomal pAQP-KO, the overall GFP-intensity distribution was almost identical to that of parasites that had been transfected with twice digested pAQP-KO.

Since eliminating episomally transfected parasites by gating appears impossible, we propose to eliminate any potential risk of generating episomally transfected mutant parasites by digesting plasmid DNA twice. To demonstrate the efficiency of linearization of plasmid DNA, competent *Escherichia coli* XL1-Blue cells were transformed with transfection DNA subjected to different restriction digestion protocols (Fig. 2B). Significant amounts of undigested plasmid remain after a standard overnight restriction digestion. In contrast, very little undigested plasmid DNA remained as indicated by a *E. coli* transformation efficiency of 0.1–0.2% of undigested DNA control, after either (i) addition of extra restriction endonuclease for the final 3 h, or (ii) when a different restriction endonuclease was added either overnight or for the final 3 h.

### 3.3 *Plasmodium* AQP is a type II aquaporin with abnormal features

**Supplementary Figure 1:**
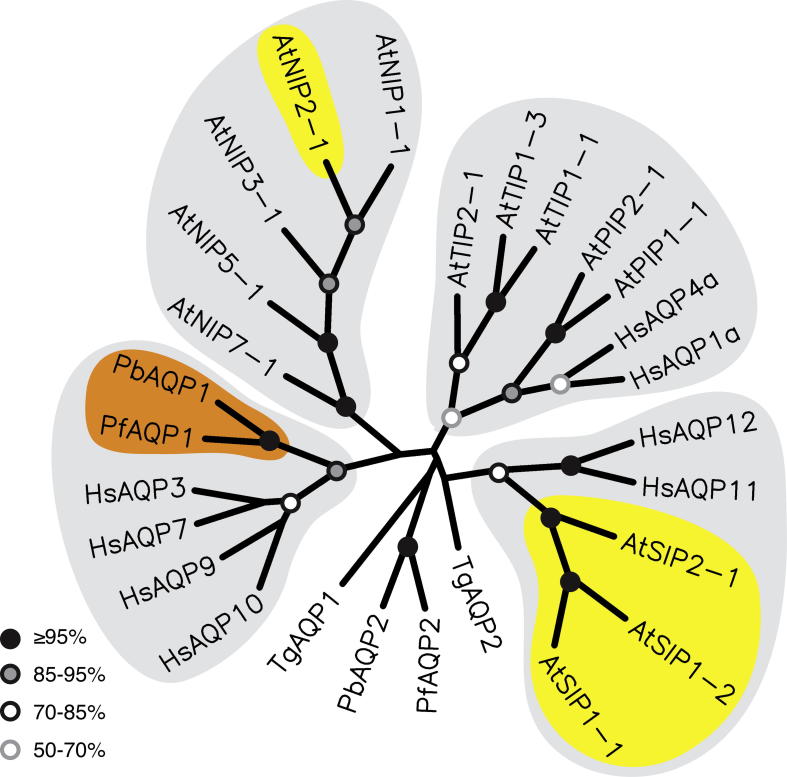


To validate the proposed flow-cytometric isolation of isogenic mutant parasite lines, two recombinant parasite lines were generated, where *AQP* was deleted or tagged with a fluorescent fusion protein. *Plasmodium* spp. and *Toxoplasma gondii* harbor two predicted aquaporins each (Supplementary Fig. S1). Phylogenetic analysis, in comparison with representative AQPs of human and *Arabidopsis thaliana,* revealed a strong association of *Plasmodium* AQP (**PBANKA_091560**; **PF3D7_1132800**) with human type II aquaporins and, more distantly, with the *A. thaliana* nodulin26-like intrinsic protein (NIP) family (Supplementary Fig. S1). *Toxoplasma gondii* AQP1 (**TGME49_015450**) clusters with the second, unusually long, predicted *Plasmodium AQP* (**PBANKA_142710**; **PF3D7_0810400**) and associates more closely to a clade containing the *A. thaliana* small basic intrinsic protein (SIP) family members and human AQP11 and 12.

Aquaporins are characterized by two “NPA” boxes that are relatively well conserved across most subfamilies (Ishibashi, 2006). Sequence alignments of these motifs of *P. berghei* and *P. falciparum* AQP1 with selected aquaporin sequences, i.e. human AQP3, 7, 9, and 10 and the *A. thaliana* NIP subfamily members, reveals significant conservation but also some striking differences (Supplementary Fig. S2). *Pb*AQP1 and *Pf*AQP1 are both marked by unusual variations in the invariant “NPA” sequences. Furthermore, a highly conserved proline in the second “NPA” box is absent in both malaria parasites.

### 3.4 AQP is dispensable under normal conditions

**Fig. 3:**
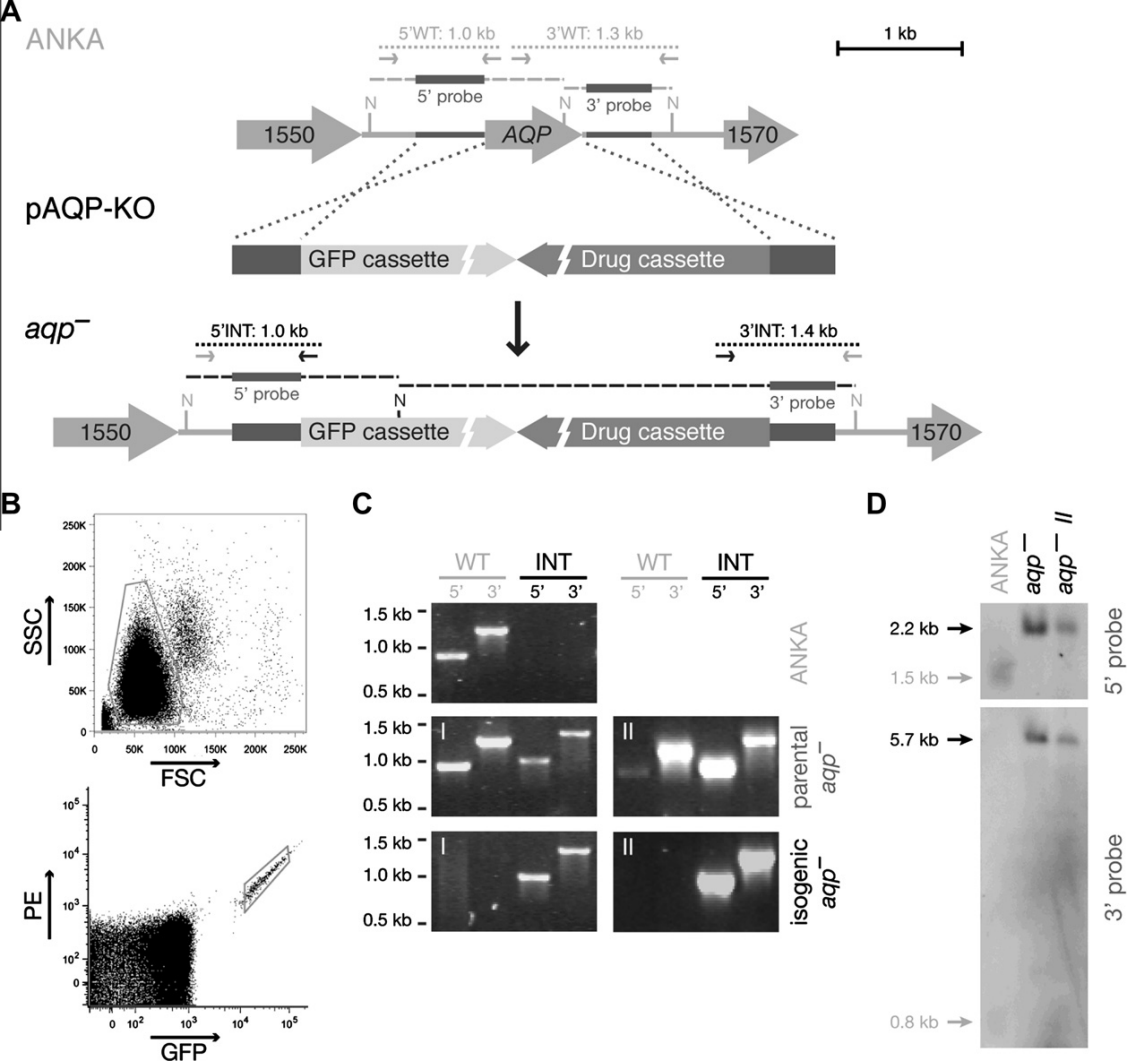
Generation and genotypic verification of *P. berghei aqp*^−^ lines. (A) Schematic representation of the generation of *aqp*^−^ parasites. The aquaglyceroporin gene *(AQP)* is located on *P. berghei* chromosome 9 between PBANKA_091550 and PBANKA_091570 (gene numbers 1550 and 1570, respectively). The 5′ and 3′ untranslated region (UTR) sequences of *AQP* were cloned into the pBAT-SIL6 *P. berghei* transfection vector (pAQP-KO). Using a double crossover/ends-out homologous recombination strategy, *AQP* was replaced with the GFP and drug-selectable cassettes. (B) Scatter plots of a parental *aqp*^−^ population following transfection of wild type (WT) (ANKA) parasites with pAQP-KO. The erythrocyte population was first gated using the side scatter (SSC) versus forward scatter (FSC) plot (as indicated by the gray box) to exclude small debris and large cells. Fifty GFP^hi^ cells were isolated from this population using a strict gate (small gray box), excluding autofluorescent cells with equal GFP and phycoerythrin (PE) levels, and were injected i.v. into naïve recipient mice. (C) Diagnostic PCR as indicated in (A) was used to demonstrate successful integration (INT) of pAQP-KO in parental *aqp*^−^ lines from two independent transfection experiments and absence of WT contamination following sorting of the isogenic *aqp*^−^ lines. (D) Southern blot analysis of both independent isogenic *aqp*^−^ lines. The 5′ and 3′ probes as indicated in (A) were used to hybridize *Nde*I restriction-digested genomic DNA of WT and knockout (KO) parasites.

A targeting vector was generated, pAQP-KO, to deplete *AQP* by double crossover/ends-out homologous recombination (Fig. 3A). In two independent transfection experiments, *aqp*^−^ parasites were readily generated (Fig. 3C, D). Before mice reached 1% parasitemia (days 7–9 after transfection), tail blood was collected for flow cytometry-assisted cell sorting (Fig. 3B) and 50 GFP^hi^ cells were injected i.v. into one naïve recipient mouse each. As expected (Fig. 1), the isogenic *aqp*^−^ parasite populations were devoid of any WT parasite contamination (Fig. 3C, D) and were used directly for subsequent analysis of *aqp*^−^ life cycle progression.

**Fig. 4:**
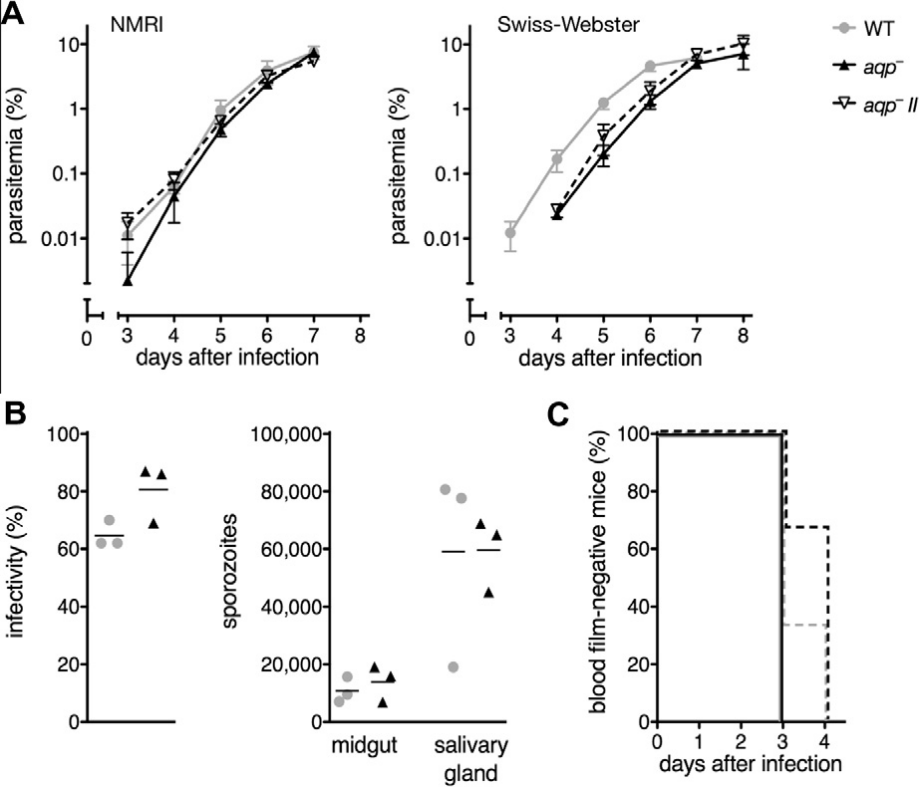
Life cycle progression of *P. berghei aqp*^−^ parasites is not significantly affected. (A) Blood stage growth in NMRI and Swiss-Webster mice. Two independent *aqp*^−^ parasite lines (black triangles) and wild type (WT) parasites (GFPhigh; gray circles) were used to infect mice. Shown are the means (±S.D.) of three replicate experiments (*n *= 3 each). Blood stage development in Swiss-Webster mice is shown as means (±S.D.) from a single experiment (WT, *n *= 4; *aqp*^−^, *n *= 3; *aqp*^−^*II*, *n *= 3). *P > *0.05; one-tailed student’s *T* test. (B) Percentage of infected mosquitos and numbers of midgut- and salivary gland-associated sporozoites in *aqp*^−^ (black triangles) and WT (GFPcon; gray circles) parasites. Data are from three independent feeding experiments. (C) Kaplan–Meier analysis of infection (*n *= 3 each) by natural bite of 12 infected mosquitos (solid lines) and by i.v. injection of 10,000 salivary gland-associated sporozoites (dashed lines) using *aqp*^−^ (black) and WT (GFPcon; gray) parasites.

No significant difference in blood stage development was observed in NMRI mice of *aqp^-^* parasites compared with a reference line (GFPhigh; Fig. 4A). In an earlier study, a significant growth delay was reported when *aqp*^−^ parasites were grown in Swiss-Webster mice (Promeneur et al., 2007). Therefore, the analysis was repeated in this mouse strain, demonstrating a detectable yet non-significant delay in both independent *aqp*^−^ lines compared with WT parasites (Fig. 4A). For analysis of life cycle progression, *aqp*^−^ parasites were transmitted through mosquitoes side-by-side with a reference line (GFPcon) (Janse et al., 2006a) and parasite numbers after host switches were monitored (Fig. 4B, C). No obvious difference was observed in exflagellation activity of *aqp*^−^ parasites compared with GFPcon parasites (data not shown). Similarly, there were no differences in mosquito infectivity, or in midgut- or salivary gland-associated sporozoite numbers (Fig. 4B). Furthermore, transmission of malaria parasites to mice either through natural bite of 12 infected mosquitoes or through i.v. injection of 10,000 isolated salivary gland sporozoites was also unaffected (Fig. 4C). We conclude that *PbAQP* does not perform vital functions for *Plasmodium* life cycle progression in vivo, at least under physiological conditions.

### 3.5 AQP is expressed in all blood stages and localizes to structures inside the parasite

**Fig. 5:**
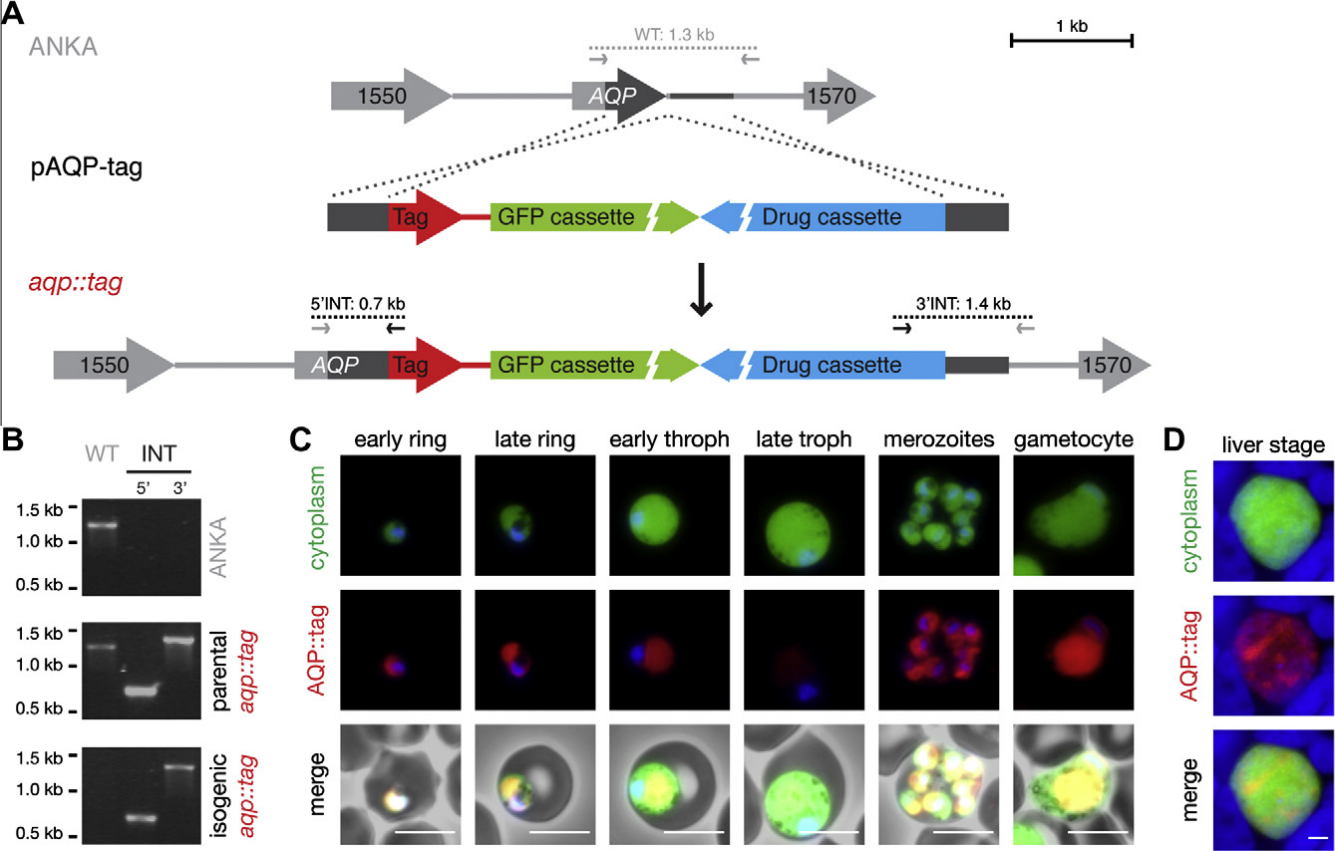
Aquaglyceroporin (AQP) is predominantly expressed during asexual blood stage development and has a perinuclear localization within the *Plasmodium berghei* parasite. (A) Schematic representation of the generation of *aqp::tag* parasites. The C-terminal and 3′ untranslated region (UTR) sequences of *AQP* were cloned into the pBAT-SIL6 *P. berghei* transfection vector (pAQP-tag). Using a double crossover/ends-out homologous recombination strategy, *AQP* was fused in-frame to an mCherry-3xMyc tag, simultaneously introducing the GFP and drug-selectable cassettes. (B) Diagnostic PCR as indicated in (A) verifies successful integration of pAQP-tag in the parental *aqp::tag* line and absence of wild type (WT) contamination following sorting of the isogenic *aqp::tag* line. (C) Live fluorescent imaging of *aqp::tag* blood stage parasites reveals the expression of AQP::tag in asexual and sexual blood stages. Note the relatively weak signal in maturing trophozoites and the well-defined perinuclear localization within the parasite. Bars, 5 μm. (D) Immunofluorescence using c-myc antibodies demonstrated expression of AQP::tag in fixed liver stage parasites 48 h p.i. Bar, 5 μm.

In addition to the null-mutant, a transgenic parasite line was generated, where AQP was fused in-frame to the red fluorescent protein mCherry, followed by a triple c-myc epitope tag. The *aqp::tag* parasites were generated using a strategy equivalent to the generation of *aqp*^−^ parasite lines (Fig. 5A). The pAQP-tag construct was readily integrated into the parental parasite population and an isogenic line expressing an AQP::tag fusion protein was isolated using flow cytometry and i.v. injection into a single mouse, without any WT contamination (Fig. 5B).

Live imaging of AQP::tag parasites revealed ample *Pb*AQP expression in asexual and sexual blood stage parasites (Fig. 5C). Reduced expression in late trophozoites correlates with reduced steady state mRNA levels for *AQP* (Hoeijmakers et al., unpublished data). In *P. falciparum*, *AQP* transcription levels increase strongly during intra-erythrocytic growth reaching their maximum in mature schizonts (Le Roch et al., 2003). The mCherry staining pattern is consistent with structures inside the parasite cytoplasm, as indicated by the GFP staining. In many cases, AQP::tag was localized in a well-defined rim surrounding the nucleus reminiscent of the endoplasmic reticulum. Immunofluorescent staining with an mCherry antibody on liver stage parasites fixed at 48 h after infection demonstrated the expression of AQP::tag and confirmed the localization inside the parasite cytoplasm (Fig. 5D).

## 4 Discussion

The optimized flow-cytometric methodology presented here allowed the rapid generation and isolation of two isogenic gene KO lines and one isogenic, tagged parasite line with the use of only six mice following the transfection. In contrast, conventional in vivo cloning through limiting dilution would have required at least 33 mice. Hence, by choosing to study isogenic populations and using flow cytometry-assisted cell sorting to isolate these, it was possible to reduce the use of animals for the generation of mutant parasite lines by >80%.

Parasites expressing high levels of GFP under control of the *HSP70* promoter were isolated reliably even in the presence of 100-fold WT parasite excess and under most conditions tested. While under the control of the stronger *HSP70* promoter, instead of the previously used *EF1α* promoter, the GFP^hi^ cell population separated more distinctly from non-fluorescent WT parasite-infected cells thus significantly reducing contamination. Furthermore, the original protocol described by Janse et al., (2006b) used flow cytometry-assisted sorting as a means of selecting successfully transfected parasites. By using drug-induced selection with pyrimethamine, parental parasite populations were enriched with successfully recombineered parasites prior to the flow cytometry-assisted isolation of these mutant parasites.

To prevent contamination with double-infected cells harboring WT and GFP^hi^ parasites, we recommend that flow cytometry is undertaken when parasitemias are still below 1%. To avoid unnecessarily long sorting periods that might damage sorted parasites, the parasitemia should ideally be above 0.1%. These recommendations perfectly match the ideal starting parasitemias for the classical in vivo cloning (Janse et al., 2006a). Furthermore, careful selection of the sorting gate, i.e. around the center of the GFP^hi^ cell population, omitting the top and bottom 20%, might even further reduce the potential risk of sorting non-fluorescent parasites or parasites propagating episomal plasmid copies.

It is important to emphasize that the approach described herein generates genetically homogenous, so-called isogenic, parasite populations and not clonal parasite lines. Paradoxically, an important potential advantage for using isogenic over clonal parasites is the potentially increased variation in the genetic background that might evolve following the transfection procedure. Consequently, an isogenic population demonstrating a distinct phenotype may be considered more robust than a clonal line when analyzing phenotypes such as growth rate. Alternatively, flow-cytometric purification of isogenic populations provides a means to generate a pure, recombinant parasite population that might subsequently be cloned using far fewer mice (e.g. three instead of 10) than cloning strategies from populations containing WT contamination that are normally generated during transfection. Furthermore, the flow-cytometric isolation described here could conceivably be used to isolate mutant parasites that would otherwise be outgrown by any WT background, such as parasites with a slow blood stage growth or reduced erythrocyte invasion.

Systematic phenotyping of the exemplar parasite population, *aqp*^−^*,* revealed a number of unexpected results*.* In a previous study, the authors claimed that ‘PbAQP plays an important role in the blood-stage development of the rodent malaria parasite during infection in mice and could be added to the list of targets for the design of antimalarial drugs’ (Promeneur et al., 2007). Data presented in this study do not support this view and show that absence of *PbAQP* does not significantly affect blood infection in two parasite–host combinations tested, i.e. *P. berghei* ANKA and female outbred NMRI or Swiss-Webster mice (Fig. 4A). It would follow that molecular genetics in the murine malaria model exclude candidacy of AQP as a promising anti-malarial drug target.

An important functional criterion for transport and channel proteins is their subcellular localization. From experiments using specific antisera, *Pb*AQP was interpreted as localized to the parasite plasma membrane (Promeneur et al., 2007), a notion that was included in the available literature (Martin et al., 2009). The two published immunofluorescent images of a ring stage parasite and a mature schizont (Promeneur et al., 2007), however, are inconclusive and do not support this interpretation. Instead, a perinuclear localization seems more plausible. This interpretation is consistent with another localization study that indicated an intracellular and patchy localization (Hansen et al., 2002). This apparent discrepancy was addressed by an independent approach where the endogenous AQP was tagged with a fluorescent mCherry tag (Fig. 5). Live imaging of AQP::tag parasites unequivocally shows a localization of the channel inside the parasite, in a disperse pattern surrounding the parasite nucleus, which is in good agreement with our interpretation of published data (Hansen et al., 2002; Promeneur et al., 2007). In *A. thaliana*, the AQP family also referred to as the major intrinsic protein (MIP) family consists of 35 members belonging to four subfamilies (Johanson et al., 2001). Members of the two subfamilies that associate most closely to the apicomplexan AQPs (Supplementary Fig. S1), the NIPs and SIPs, have diverse subcellular localization including the endoplasmic reticulum (Ishikawa et al., 2005; Mizutani et al., 2006). *Tg*AQP1, which displays similarity to the second predicted *Plasmodium* AQP (Supplementary Fig. S1), has been localized to a novel organelle resembling the plant vacuole in free tachyzoites (Miranda et al., 2010). Similarly, in the kinetoplastid pathogen, *Leishmania donovani*, two out of four aquaporins are found in intracellular organelles (Biyani et al., 2011), providing another example of the presence of subcellular aquaporins in protists. These data show that systematic tagging of *Plasmodium* proteins through rapid, flow-cytometric isolation of fluorescently labeled recombinant parasites might aid in prioritizing the need to generate specific antisera on a protein-by-protein basis.

A similar delay in *P. berghei* blood stage infection of Swiss-Webster mice, as seen for the *aqp*^−^ parasite line (Promeneur et al., 2007), was observed when WT *P. berghei* parasites were grown in *aqp9*^−^*^/^*^−^ mice (Liu et al., 2007). To date, it remains unclear whether the lack of both host and parasite *AQP* leads to a synergistic effect or if both AQPs function in a cascade where the disruption of a single channel protein achieves the maximum effect. Glycerol uptake in mouse erythrocytes is mediated through AQP9 (Liu et al., 2007); likewise AQP3 is responsible for glycerol uptake in human and rat erythrocytes. Interestingly, human AQP3 appears to be recruited to the parasite vacuolar membrane in human erythrocytes infected with *P. falciparum* (Bietz et al., 2009), which could hint at a cascade of host- and parasite-derived AQPs functioning together in the growing parasite for solute (water and glycerol) import. Furthermore, if malaria parasites indeed recruit host cell AQP, this could minimize the need for the export of parasite-derived AQP.

In this context, the observation by Promeneur et al. (2007) that *aqp*^−^ null mutant infected erythrocytes show a marked decrease in the uptake of radiolabelled glycerol compared with WT-infected erythrocytes is puzzling. One plausible explanation is that in the absence of *Pb*AQP, glycerol and, perhaps, other solutes cannot accumulate in an intracellular storage organelle, such as the endoplasmic reticulum, and instead accumulate in the parasite cytoplasm. As a consequence of altered glycerol concentrations in the cytoplasm and/or certain specific subcellular compartments, pathways controlling extracellular glycerol uptake may no longer get activated. Candidate proteins involved in this uptake include the second *Plasmodium* aquaporin (Supplementary Fig. S1), or host recruited AQP (Bietz et al., 2009). A second explanation could be a possible dual localization of *Pb*AQP1, which the data presented here cannot exclude entirely.

Molecular genetics in *P. berghei*, the most advanced murine malaria model, can provide invaluable insights into the molecular functions of individual parasite genes and provide tools to study the intimate parasite-host interactions at various stages during the complex life cycle. Our long-term vision is a genome-scale repository of mutant and transgenic malaria parasite lines that serves the research community to focus on the scientific questions rather than spending resources and often parallel efforts in order to generate the necessary tools. We believe that rapid isolation of isogenic recombinant *P. berghei* parasites reported herein overcomes or mitigates a major consideration, that is the extensive use of animals for in vivo cloning, towards achieving that goal.
