# Supplementary material for: Flow cytometry-assisted rapid isolation of recombinant Plasmodium berghei parasites exemplified by functional analysis of aquaglyceroporin
Source: Int J Parasitol. 2012 Dec;42(13-14):1185–92. doi: 10.1016/j.ijpara.2012.10.006 (PMC3521960; doi:10.1016/j.ijpara.2012.10.006)
Supplement: Supplementary data 1 [file mmc1.docx]

| **Supplementary Table S1.** Summary of primer sequences used in this study. | |
| --- | --- |
| **Primer Name** | **Primer Sequence (restriction sites are underlined)** |
| AQP-F1 | CTTTACCCCCTATTTAACATATGATG |
| AQP-F2-*Sac*II | AAACCGCGGCTTAAAAAGTATAAATGACTGTAAAAGAG |
| AQP-F3 | TGTTACCGTGGGGTTATCAACC |
| AQP-F4-*Sac*II | TAACCGCGGTATTTTGCAGGACAACTCCTTG |
| AQP-F5-*Avr*II | AATCCTAGGCATTATTATAAAATATATTTGTGAGACTG |
| AQP-R1-*Eco*RI | AAAGAATTCTTAATTAAGATATTTTTCTTTTGAAAAAAAATAAAATAATATATATAA |
| AQP-R2 | AATCGTTTTTAATCGGGACAGC |
| AQP-R3-*Psh*AI | TAAGACATATGTCCTATTTCTAAGGCGCCTTTATCATG |
| AQP-R4-*Kpn*I | ATAGGTACCTTGCAAATCTGTCTTGTCGTC |
| AQP-R5 | CTGTTCGCTTCATATCCATACC |
| SIL6F | GACAGCGCATATGATGGATG |
| SIL6R | TACGAATACGCAATTTCTCAAAC |
| 5’HSP70rev | CAATTTGTTGTACATAAAATAGGCAG |
| 5’DHFRrev | ATGAAATACCGCTCCATTTTTCC |
| mCherryRev | CCCTCCATGTGAACCTTGAAG |
