# Supplementary material for: Flow cytometry-assisted rapid isolation of recombinant Plasmodium berghei parasites exemplified by functional analysis of aquaglyceroporin
Source: Int J Parasitol. 2012 Dec;42(13-14):1185–92. doi: 10.1016/j.ijpara.2012.10.006 (PMC3521960; doi:10.1016/j.ijpara.2012.10.006)
Supplement: Supplementary data 2 [file mmc2.docx]

PbAQP1 ISAKLSGAHLNLAVTVGLSTIKK … SFGGNTGFALNPSRDLGARILSA

PfAQP1 VSAKLSGAHLNLAVSIGLSSINK … TFGGNTGFALNPSRDLGSRFLSL

**amino acid**

HsAQP3 IAGQVSGAHLNPAVTFAMCFLAR … SMGFNSGYAVNPARDFGPRLFTA **types**

HsAQP7 VAGRISGAHMNAAVTFANCALGR … SLGMNTGYAINPSRDLPPRIFTF ————————————

HsAQP9 VAGGVSGGHINPAVSLAMCLFGR … SLGLNSGCAMNPARDLSPRLFTA  **a**liphatic

HsAQP10 VGGNVSGAHLNPAFSLAMCIVGR … SMGANCGIPLNPARDLGPRLFTY hydrophi**l**ic

hydropho**b**ic

AtNIP1-1 SLGHISGAHINPAVTIAFASCGR … IAAPVSSASMNPGRSLGPALVYG  **n**egative

AtNIP2-1 CLGHLS-AHFNPAVTLALASSQR … FAGEVSGASMNPARSIGPALVWG NH**2**

AtNIP3-1 SIGHVSGAHFNPAVSIAFASSKK … FSGPISGASMNPARSLGPALIWG **O**H

AtNIP5-1 STGHISGAHLNPSLTIAFAALRH … VAGPSTGGSMNPVRTLGPAVASG **p**ositive

AtNIP7-1 SIGHISGAHLNPSITIAFAVFGG … ITGPISGGSMNPARSLGPAVVAW **t**iny

**X** identical

type attlaSGtHbN tbObtbtOblp … Obt 2Ot tbNPtRnbt pbbOa amino acid
